# Stress on facial skin of class III subjects during maxillary protraction: a finite element analysis

**DOI:** 10.1186/s12903-019-0724-6

**Published:** 2019-02-13

**Authors:** Francesca Gazzani, Chiara Pavoni, Paola Cozza, Roberta Lione

**Affiliations:** 1 Department of Clinical Sciences and Translational Medicine, University of Rome ‘Tor Vergata’, Via Collazia 29, 00183 Rome, Italy; 2 Department of Dentistry UNSBC, Tirana, Albania

**Keywords:** FEM analysis, Face mask, Maxillary protraction, Facial skin stresses

## Abstract

**Background:**

Maxillary protraction with facemask (FM) is an orthopedic approach for treatment of Class III growing patients. Aim of the present investigation was to analyze tension loads produced by two different facial mask (FM) designs on facial skin of subject with skeletal Class III.

**Methods:**

A three-dimensional (3D) geometry of Delaire and Petit FM models were reconstructed from the original Computer Aided Design (CAD) 3D prototype using software package (ANSYS 5.7). A traction load of 9.8 N inclined of 30° to the occlusal plane was applied combining analytical FM models with a 3D facial model. Resulting stresses and deformations on the skin layer were tested through the von Mises yield criterion.

**Results:**

Overall tensions were mostly developed on the chin area, while lower stresses were observed on forehead area for both FM designs. When Delaire FM model was tested, maximum stresses were observed on the upper border of the chin cup corresponding to the inferior lip and to marginal gingiva of lower incisors. After Petit FM application, maximum stresses were more extensively localized at the level of both upper border and central area of the chin. Stresses measured on the chin area were significantly higher with Petit FM when compared with Delaire FM (44 KPa versus 29 KPa, respectively).

**Conclusions:**

Delaire FM determined lower stresses and tensile tensions than Petit FM model. Highest tensions were observed at the level of chin cup area for both Delaire and Petit FM. Stresses following Delaire FM application were mostly observed on the upper border of the chin cup, while Petit FM determined stresses more extensively distributed to the central area of the chin.

## Background

### Introduction

Face-mask (FM) therapy is the most recommended orthopedic approach for early treatment of skeletal Class III malocclusion [1–5]. Many articles [1–5] described favorable and stable outcomes on dento-skeletal structures and on soft tissue profile of maxillary protraction protocol. During orthopedic treatment, tensile forces are applied on maxillary sutures allowing a forward displacement of the maxilla and an improvement of the sagittal dento-skeletal relationship [6, 7]. Orthopedic therapy requires the application of heavy forces ranging from 7.8 N to 9.8 N (800–1000 g, respectively) that often cause skin irritation and even mild swelling [7, 8]. Original FM model was firstly described by Jean Delaire [9, 10] and it consists of two extra-oral plastic supports connected to a metallic framework composed by two lateral vertical bars and one cross bar. Some years later, Petit [11] introduced a modified model of Delaire FM with a single rod running in the midline between the chin and forehead plastic cups. Recently, three-dimensional (3D) Finite Element Analysis (FEA) [6, 7, 12] has been used in order to evaluate the displacement and stress distribution of orthopedic forces applied on maxillofacial structures. Gautam et al. [6] highlighted that the highest loads produced by FM therapy were mostly localized along naso-maxillary, frontonasal and, fronto-maxillary sutures. Gazzani et al. [13] evaluated mechanical properties of Delaire FM and the stresses generated on device components during orthopedic treatment, by means of FEA. Some of the major complications related to maxillary protraction treatment are due to poor adaptation and low fit of FM on the face and to skin irritation determined by the extra-oral cups. The discomfort in wearing the FM device affects patients’ compliance with negative consequences on treatment effectiveness.

### Purpose of the study

Hence, the aim of the present FEA study was to analyze tension loads produced by two FM designs and their effects on the chin and forehead skin area during orthopedic maxillary protraction.

## Methods

### Finite element analysis (FEA)

The FEA was performed defining the following parameters:

- geometrical features of both Delaire and Petit FMs;
- geometrical feature of 3D facial model prototype;
- material properties for each element of the FMs;
- support bone properties;
- mesh (number, shape and size of the elements used to discretize the FMs);
- constrains and loads applied on the system.

**Fig. 1:**
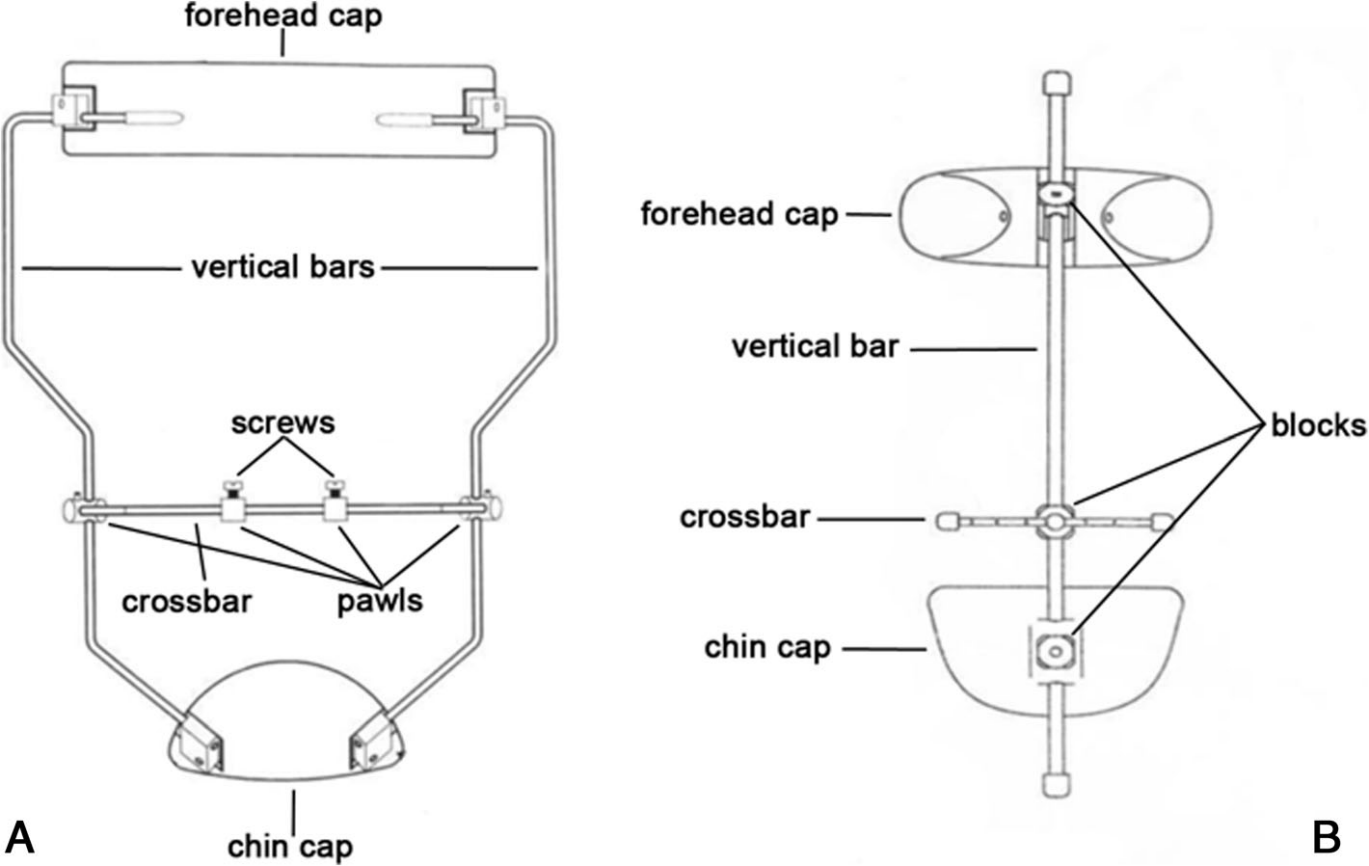
Structure and parts of Delaire FM (**a**) with two lateral bars and Petit FM (**b**) with single median bar

**Fig. 2:**
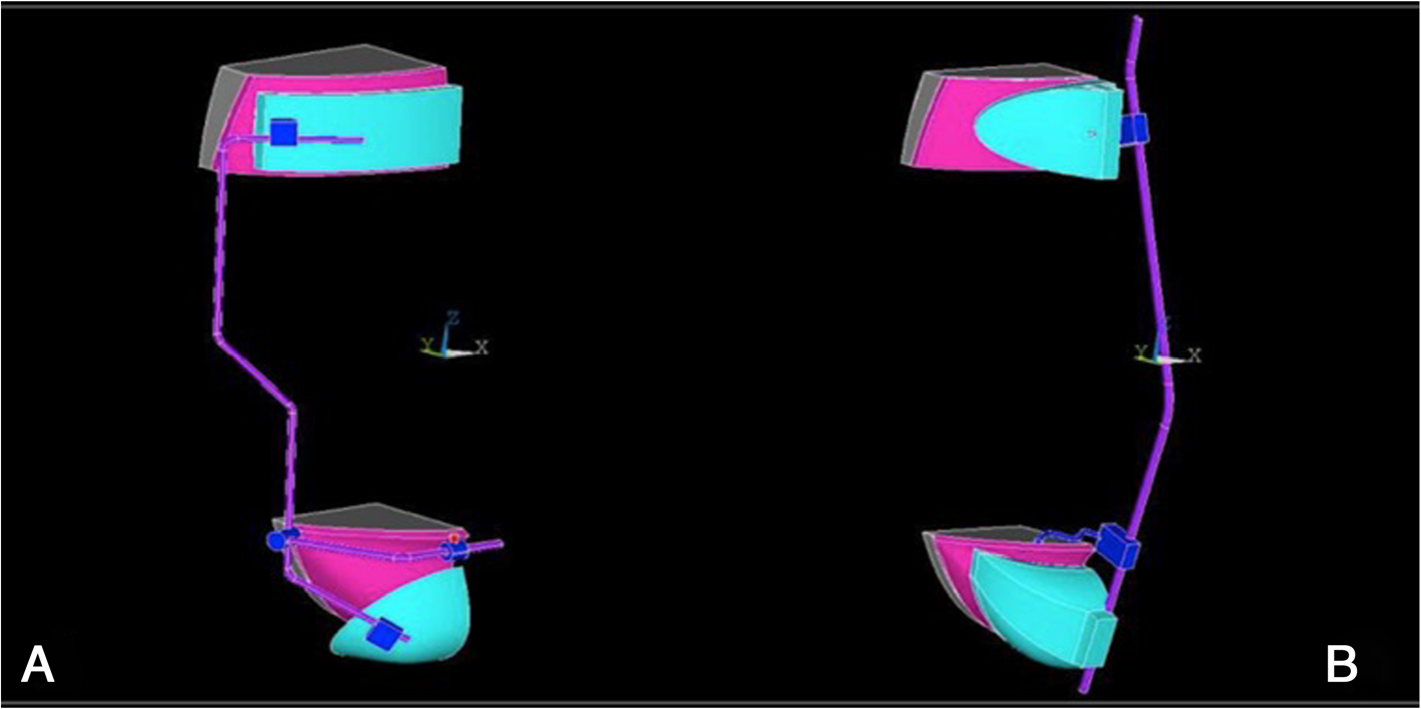
3D models of Delaire FM (**a**) and Petit FM (**b**) designed by using ANSYS 5.7

The ANSYS 5.7 software (Ansys Inc., Canonsburg, PA, USA) was used for the FEA. The software solved the steady state condition of a rigid body in the space considering the input data. In particular, the system of algebraic equations was solved iteratively until the convergence of the solution was reached. Regarding the output data, stress and strain state induced by FM rigid bodies on the facial prototype were evaluated. Two analytical models were developed by considering Delaire-FM (M0774–01 Leone S.p.A., Florence, Italy) and Petit-FM (M0772–1 Leone S.p.A., Florence, Italy) (Fig. 1). Each component of the two models was constructed and assembled using Rhinoceros 4.0 CAD software (Robert McNeel & Associates, Seattle, WA, USA) and then exported to ANSYS 14.0 (Ansys Inc., Canonsburg, PA, USA) for the FEA (Fig. 2). For the 3D facial model, a human head prototype was chosen from Rhinoceros software and then modified considering a support bone with Young’s Modulus of 18,000 MPa and a 3 mm skin layer with Young’s Modulus of 0.64 MPa [14]. However, only forehead and chin areas of the model were considered and imported in ANSYS in order to simplify the FEA. In the 3D facial model an ideal occlusal plane was simulated for force application. The occlusal plane was orientated respect the ala-tragus line considering the facial model positioned in natural head position (NHP). For both Delaire and Petit FM, a 9.8 N (4.9 N for each side) loading force was applied with a 30° downward inclination respect to the occlusal plane to replicate the clinical application of the FM [3–5, 13, 15]. The FEA static simulation was conducted applying both FM types on 3D facial model. Thus, stress and load distribution on the skin areas were evaluated by using a static load. The mesh phase and the loads were developed by means of the interactive interface of the software.

### Evaluation of load and stress distribution

In order to underline the load and stress distribution on the forehead and chin areas, von Mises yield criterion was evaluated on the skin layer delimitated externally by the FMs cups and internally by the bone structure. The amount of elastic energy absorption was calculated for 9.8 N inclined of 30° to the occlusal plane to quantify the deformation state induced on the skin areas involved by the two FM models.

## Results

**Fig. 3:**
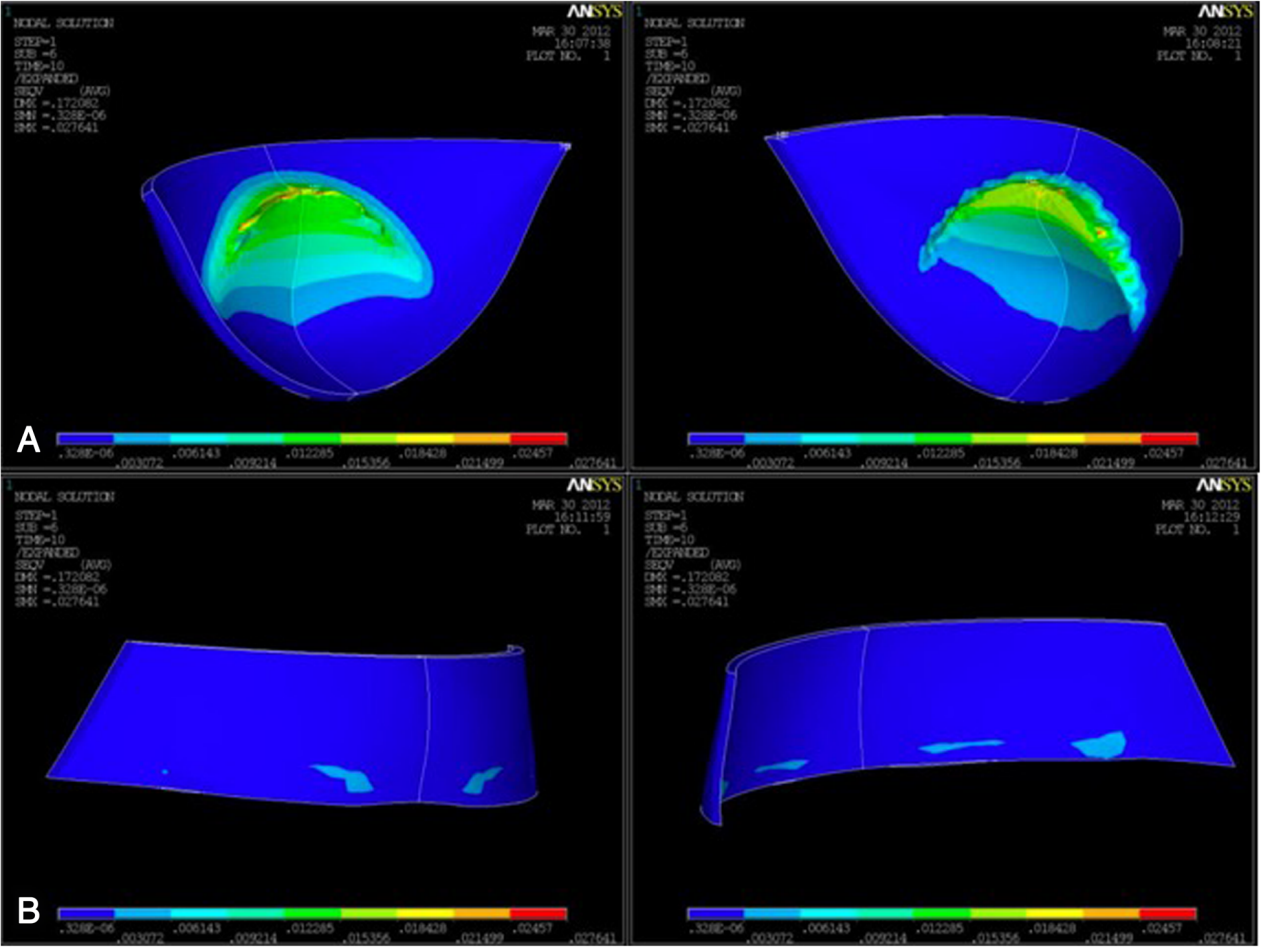
Visualization of Von Mises criterion trend by means of a color-coded map describing tensions intensity scale. Blue indicates lowest tensions, and red the highest. Color values for intermediate intensities are interpolated from cyan, green, and yellow **a.** Stress distribution on the chin area induced by Delaire FM (external and interior side). Intermediate tensions in green were observed at the level of the upper border of the chin and tended to decrease as moving away from this area. **b.** Stress distribution on the forehead area induced by Delaire FM (external and interior side)

**Fig. 4:**
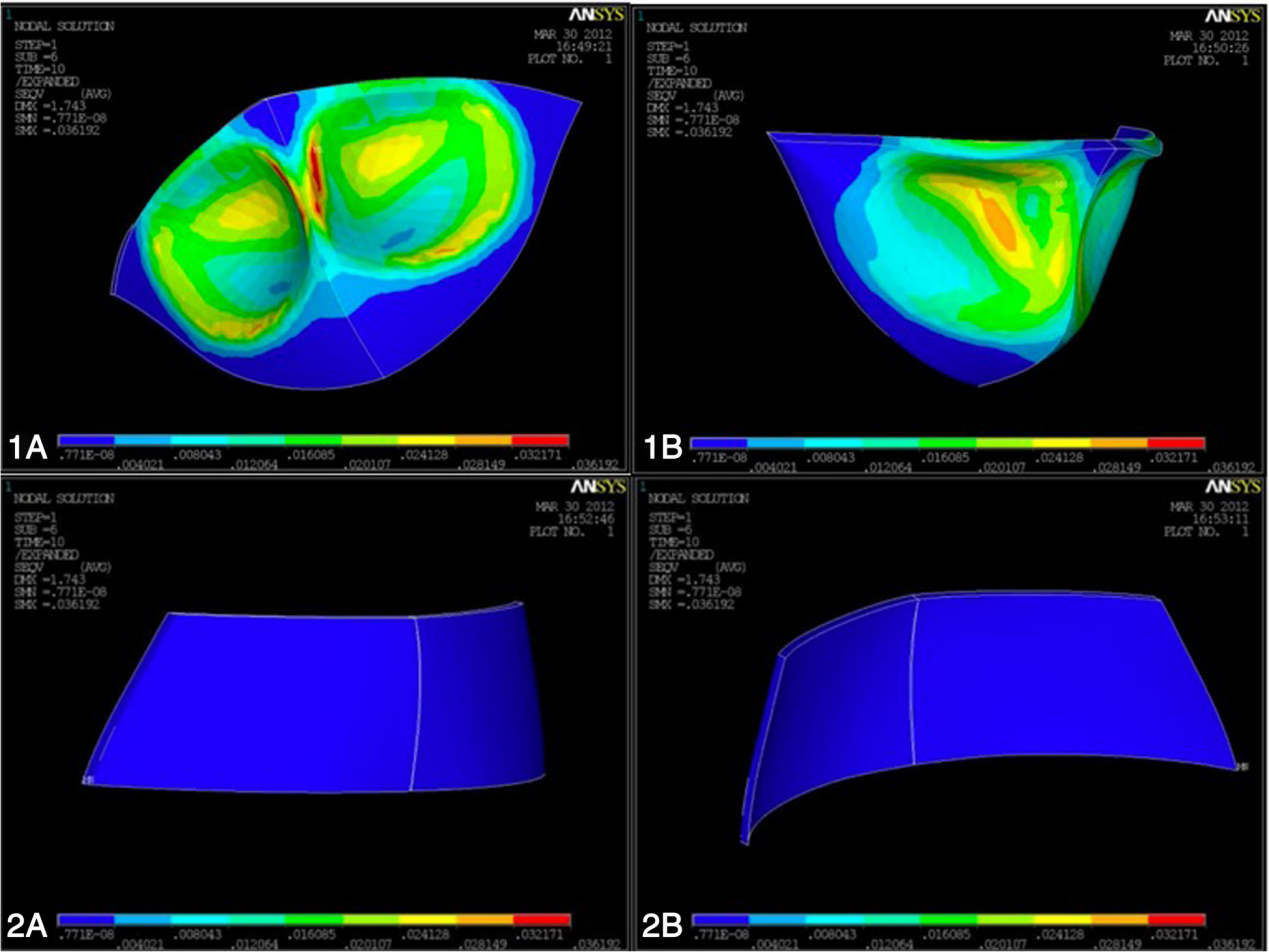
Visualization of Von Mises criterion trend by means of a color-coded map describing the tensions intensity scale. Blue indicates lowest tensions, and red the highest. Color values for intermediate intensities are interpolated from cyan, green, and yellow. **a.** Stress distribution on the chin area induced by Petit FM (external and interior side). High tensions in red and yellow were observed at the level of the upper border of the chin and tended to decrease as moving outward. **b.** Stress distribution of the forehead area induced by Petit FM (external and interior side). Homogeneous blue area with no different colors indicates the lack of tensile tensions induced by the Petit FM

The results of the von Mises yield criterion are shown in Figs. 3 and 4 and Table 1. For both FM models, greater tensions were recorded at the level of the chin cup with higher stresses and deformations observed for the Petit FM (29 KPa and 44 KPa, for Delaire and Petit FM, respectively (Table 1). When Delaire FM was tested, maximum stresses were observed on the upper border of the chin cup corresponding to the inferior lip and to marginal gingiva of lower incisors (Fig. 3A). Moreover, maximum stresses tended to decrease constantly as moving away from this area. Similarly, tensile strength distribution analysis showed maximum stresses in correspondence of chin cup area after Petit FM application (Fig. 4A). In particular, tensile tensions were more extensively distributed and localized in correspondence of both upper border and central area of the chin. Within all tests performed, lower stresses were observed in the forehead area respect to the chin area (Figs. 3B,4B). Heavier tensions developed on the forehead were at least three times lower than the maximum stresses affecting the chin (7 KPa and 3 KPa, induced by Delaire and Petit FM, respectively).

**Table 1:** Maximum tensions on the skin areas

Mask type	Max. tension on chin skin surface	Max. tension on forehead skin surface
Delaire	29 kPa	6 kPa
Petit	44 kPa	3 kPa

kPa, kilopascal. 1 kPa correspond to 1000 Pa

## Discussion

FM therapy represents the gold standard for correcting skeletal Class III malocclusion in growing subjects [1–5, 16–19]. Several factors play a substantial role in terms of efficacy and effectiveness of orthopedic Class III treatment, including individual skeletal pattern, protraction device adaptation, patients’ compliance, and device wear-time [20–24]. Stocker et al. [23] and Ozkalayci and Cicek [24] analyzed wearing time and patient compliance with FM reporting a significantly lower wearing time than the prescribed instructions. The not customized design of both FM models can cause poor device adaptation to the patient’s face. The less adhesion of the device could determine skin irritations and mild swelling, with negative effects on patient compliance [1–5]. Stresses and load distribution developed on skeletal structures during orthopedic protraction treatment were widely analyzed [6, 7, 25]. More recently, in a previous investigation stresses developed during maxillary protraction and their effects on FM structure were evaluated by means of FEM analysis [13]. The absence of permanent plastic deformations and efficacy persistence of FM components highlighted the importance of a careful management of the device during treatment in order to grant its best performance. Although adverse effects on facial skin are known and very frequent in daily practice, however no data are available in literature regarding distributions and effects of tensile forces on facial skin. To our knowledge, both Petit and Delaire FM can lead to skin irritations caused by the plastic forehead and chin pads. Plastic supports of FM should to be adjusted to fit patient’s face maximizing the contact surface with the skin for a homogeneous distribution of the loads applied and avoiding skin wounds. Overall results of the present study highlighted higher tensile tensions at the level of the chin cup after the application of the Petit FM when compared with the Delaire FM (44 kPa and 29 kPa, respectively) (Table 1). More extended chin support of Delaire design reduced the intensity of the residual tensions and stresses developed on the patient skin face, while smaller chin support of Petit FM tended to increase the tensile tensions on the face exposing the patient to more skin irritation. Lower tensile tensions decrease the risk of skin irritations, but also maximize orthopedic forces transmission to the bone structure [2]. Moreover, tensile tensions were localized on the whole chin cup area with Petit FM, while more extended chin cup pads of Delaire FM determined maximum stresses in correspondence of its upper border. In both FM models’ analysis, tensions registered on forehead region resulted particularly low and thus, clinically irrelevant, especially when Petit FM was tested. Maximum stresses ranged from 3 kPa (Petit FM) to 7 kPa (Delaire FM) and were mostly localized on the lower area, near to the eyebrows (Table 1). Color uniformity observed on the forehead support indicates the absence of significative stresses induced by applying Petit FM (Fig. 4B). Forehead caps can be considered negligible in terms of discomfort and fit if compared with the chin supports. Tensile strength analysis confirmed good adaptation for both FM designs. It can be supposed that all tensions and stresses developed during orthopedic protraction are maximized and focused on the chin area near the center of load application, especially in the Petit structure. The results of the present FEA investigation suggested that Petit FM appears clinically less comfortable than the Delaire model with a higher risk of skin irritation and mild swelling on the chin. On the other hand, a prolonged stress applied by the border of the chin cap could interfere with the periodontal health of the lower incisors [26]. An external compression on the labial mental grove exerted by the chin pad could transmit a compression on the lower incisors’ marginal gingiva with resulting processes of gingival retraction. According to the findings of the present study, FM designs with more extended chin supports should be preferred to achieve wearing comfort. As previously suggested [8] a clinician-made customization of the chin cup using a poly-vinyl siloxane could be used to uniform the contact surfaces of the orthopedic device structure improving its performance and control of the load distribution.

Limitations of the current study were due to the 3D facial model used to perform FEM analysis of forces induced by different FM models. Standardization of 3D model did not provide data about how forces distribution could change on different facial biotype. Further clinical studies will be necessary to analyzed stresses and loads distribution of orthopedic forces induced by maxillary protraction considering different facial patterns and biotypes in order to grant optimum management and customized fit of the device.

## Conclusion

Highest tensions were observed at the level of chin cup area than at the level of forehead cups for both Delaire and Petit FM. Stresses following Delaire FM application were mostly observed on the upper border of the chin cup at the level of the inferior lip and lower incisor marginal gingiva. Petit FM produced higher tensile tensions than the Delaire design on the chin cup with stresses more extensively distributed to the central area of the chin.

## Abbreviations

3D: Three Dimensional
CAD: Computer Aided Design
FEA: Finite Element Analysis
FM: Face Mask
KPa: KiloPascal
N: Newton
